# Diversity and Activity of *Lysobacter* Species from Disease Suppressive Soils

**DOI:** 10.3389/fmicb.2015.01243

**Published:** 2015-11-16

**Authors:** Ruth Gómez Expósito, Joeke Postma, Jos M. Raaijmakers, Irene De Bruijn

**Affiliations:** ^1^Department of Microbial Ecology, Netherlands Institute of Ecology (NIOO-KNAW)Wageningen, Netherlands; ^2^Laboratory of Phytopathology, Wageningen University and Research CentreWageningen, Netherlands; ^3^Plant Research International, Wageningen University and Research CentreWageningen, Netherlands

**Keywords:** *Lysobacter*, *Rhizoctonia solani*, *Beta vulgaris*, disease suppression, plant growth promotion

## Abstract

The genus *Lysobacter* includes several species that produce a range of extracellular enzymes and other metabolites with activity against bacteria, fungi, oomycetes, and nematodes. *Lysobacter* species were found to be more abundant in soil suppressive against the fungal root pathogen *Rhizoctonia solani*, but their actual role in disease suppression is still unclear. Here, the antifungal and plant growth-promoting activities of 18 *Lysobacter* strains, including 11 strains from *Rhizoctonia*-suppressive soils, were studied both *in vitro* and *in vivo*. Based on 16S rRNA sequencing, the *Lysobacter* strains from the *Rhizoctonia*-suppressive soil belonged to the four species *Lysobacter antibioticus, Lysobacter capsici, Lysobacter enzymogenes*, and *Lysobacter gummosus*. Most strains showed strong *in vitro* activity against *R. solani* and several other pathogens, including *Pythium ultimum, Aspergillus niger, Fusarium oxysporum*, and *Xanthomonas campestris*. When the *Lysobacter* strains were introduced into soil, however, no significant and consistent suppression of *R. solani* damping-off disease of sugar beet and cauliflower was observed. Subsequent bioassays further revealed that none of the *Lysobacter* strains was able to promote growth of sugar beet, cauliflower, onion, and *Arabidopsis thaliana*, either directly or via volatile compounds. The lack of *in vivo* activity is most likely attributed to poor colonization of the rhizosphere by the introduced *Lysobacter* strains. In conclusion, our results demonstrated that *Lysobacter* species have strong antagonistic activities against a range of pathogens, making them an important source for putative new enzymes and antimicrobial compounds. However, their potential role in *R. solani* disease suppressive soil could not be confirmed. In-depth omics'–based analyses will be needed to shed more light on the potential contribution of *Lysobacter* species to the collective activities of microbial consortia in disease suppressive soils.

## Introduction

*Lysobacter* are Gram-negative bacteria widely distributed in diverse ecosystems, including soil, rhizosphere, and freshwater habitats (Reichenbach, 2006). The genus *Lysobacter* was first described in 1978 by Christensen and Cook and included four species. *Lysobacter* spp. are closely related to members of the genus *Xanthomonas* and were initially misclassified as *Cytophaga, Sorangium*, or *Myxobacter* (Christensen and Cook, 1978). Currently, 30 *Lysobacter* species have been taxonomically accepted (for updates see http://www.bacterio.net/lysobacter.html) and new *Lysobacter* species have been recently identified (Du et al., 2015; Lin et al., 2015; Singh et al., 2015) but are not yet included in the database. Various members of this bacterial genus have activity against a range of other (micro)organisms, including Gram-negative and Gram-positive bacteria, fungi, oomycetes, and nematodes (Reichenbach, 2006). They are well-known for the production of a variety of extracellular enzymes and antimicrobial compounds. Enzymes identified for *Lysobacter* include chitinases (Zhang and Yuen, 2000; Zhang et al., 2001), glucanases (Palumbo et al., 2005), proteases (Stepnaya et al., 2008; Gökçen et al., 2014; Vasilyeva et al., 2014), lipases (Folman et al., 2003; Ko et al., 2009) as well as elastases, keratinases, phosphatases, endonucleases, endoamylases, and esterases (Reichenbach, 2006). Antimicrobial compounds described for *Lysobacter* include lysobactin, tripopeptin, xanthobaccin, maltophilin, dihydromaltophilin, phenazine, lactivicin (Xie et al., 2012), HSAF (Li et al., 2008), and WAP-8294A2 (Zhang et al., 2011). Currently, WAP-8294A2 is in phase I/II clinical trials for controlling methicillin-resistant *Staphylococcus aureus* (anti-MRSA) (Zhang et al., 2011; Wang et al., 2013).

In terms of ecosystem services, Postma et al. (2010a) showed a correlation between the abundance of three *Lysobacter* species (*Lysobacter antibioticus, Lysobacter capsici*, and *Lysobacter gummosus*) in soil and the level of suppressiveness against *Rhizoctonia solani*, a devastating fungal pathogen of numerous economically important crops such as sugar beet, potato, and rice. Also in the study by Mendes et al. (2011), the Xanthomonadaceae family, to which *Lysobacter* belongs, was found more abundant in a soil suppressive against *R. solani* on sugar beet. Several studies have shown that application of *Lysobacter* spp. reduced diseases caused by different plant pathogens in several crops such as cucumber (Folman et al., 2004; Postma et al., 2009), bean (Yuen et al., 2001), rice (Ji et al., 2008), pepper (Ko et al., 2009), grapevine (Puopolo et al., 2014), sugar beet, spinach (Islam et al., 2005), and tomato (Puopolo et al., 2010). To date, however, few data are available on the frequency and diversity of *Lysobacter* species in natural habitats and little is known about the ecology and the determinative role of *Lysobacter* species in plant growth promotion and disease suppressive soils.

The work described here focused on elucidating the role of *Lysobacter* spp. in protecting plants against soil-borne diseases and in stimulating plant growth. To that end, we determined (i) the genetic and phenotypic diversity of 18 different *Lysobacter* strains obtained from soil and plant-associated environments, (ii) their activity against a range of pathogens, (iii) if these *Lysobacter* strains alone can suppress damping-off disease of sugar beet and cauliflower caused by *R. solani*, and (iv) if *Lysobacter* can promote plant growth via direct contact and/or via production of volatile compounds.

## Materials and methods

### Strains, culture, and storage conditions

The *Lysobacter* strains used in this study (Table 1) were isolated from different Dutch soils suppressive to *R. solani*. Reference strains (Table 1) were obtained from the DSM strain collection (Leibniz Institute DSMZ-German Collection of Microorganisms and Cell Cultures, Braunschweig, Germany). For the activity and plant growth promotion assays, *Lysobacter* strains were pre-cultured in tryptone soya broth (TSB, Oxoid) for 2–3 days at 25°C on a rotary shaker at 200 rpm and cells were washed 3 times with 0.9% NaCl unless mentioned otherwise. The fungal pathogens used in this study were mostly provided by the Institute of Sugar Beet Research (IRS). *Fusarium oxysporum* Forl1 was provided by the University of Turin, Italy (Clematis et al., 2009), *Verticillium dahliae* JR2 by B. Thomma [Wageningen University (WUR)], *Phytophthora infestans* by F. Govers (WUR), and *Aspergillus niger* was provided by L. de Graaf (WUR) (Table S1). The bacterial strains were kept in 40% (v/v) glycerol at −80°C; the fungi and oomycetes were kept in mineral oil at 10°C.

**Table 1:** **Isolation details of the *Lysobacter* strains used in this study**.

**Code**	**Species**	**Strain**	**Soil type**	**Source**	**Crop**	**Origin**	**Location**	**Year**	**References**
L02	*Lysobacter antibioticus*	3.2.10	Clay	Soil	Grass/clover	Suppressive soil	Pietersbierum, NL	2003	Postma et al., 2008
L08	*Lysobacter antibioticus*	76	Clay	Soil	Cauliflower	Suppressive soil	Zwaagdijk, NL	2003	Postma et al., 2010b
L23	*Lysobacter antibioticus*	4.1.2	Clay	Soil	Potato	Suppressive soil	Marknesse, NL	2006	Postma et al., 2008
L32	*Lysobacter antibioticus*	DSM2044	N.A.	Soil	N.A.	Type strain	Ottawa, CA	N.A.	Christensen and Cook, 1978
173	*Lysobacter antibioticus*	173	Clay	Soil	No crop	Suppressive soil	Zwaagdijk, NL	2011	(This study)
174	*Lysobacter antibioticus*	174	Clay	Soil	No crop	Suppressive soil	Zwaagdijk, NL	2011	(This study)
L12	*Lysobacter capsici*	6.2.3	Clay	Soil	Grass/clover	Suppressive soil	Hoensbroek, NL	2003	Postma et al., 2010a
L13	*Lysobacter capsici*	1.3.3	Clay	Soil	Grass/clover	Suppressive soil	Strijen, NL	2003	Postma et al., 2010a
L14	*Lysobacter capsici*	55	Clay	Soil	Cauliflower	Suppressive soil	Zwaagdijk, NL	2003	Postma et al., 2010a
L31	*Lysobacter capsici*	DSM19286	N.A.	Rhizosphere	Pepper	Type strain	South Korea	2003	Park et al., 2008
L19	*Lysobacter enzymogenes*	1.1.4	Sand	Soil	Grass	Suppressive soil	Bakel, NL	2004	Nijhuis et al., 2010
L28	*Lysobacter enzymogenes*	3.1T8	Rockwool	Root tip	Cucumber	Rockwool	Wageningen, NL	1997	Folman et al., 2003
L29	*Lysobacter enzymogenes*	C3	N.A.	Leaf	Turfgrass	Suppressive soil	Nebraska, USA	N.A.	Sullivan et al., 2003
L30	*Lysobacter enzymogenes*	DSM2043	N.A.	Soil	N.A.	Type strain	Ottawa, CA	N.A.	Christensen and Cook, 1978
L05	*Lysobacter gummosus*	2.4.7	Clay	Soil	Grass/clover	Suppressive soil	Ijzendijke, NL	2003	Postma et al., 2008
L15	*Lysobacter gummosus*	3.2.11	Clay	Soil	Grass/clover	Suppressive soil	Pietersbierum, NL	2003	Postma et al., 2008
L26	*Lysobacter gummosus*	10.1.1	Clay	Soil	pea	Suppressive soil	Ijzendijke, NL	2006	Postma et al., 2008
L33	*Lysobacter gummosus*	DSM6980	N.A.	Soil	N.A.	Type strain	Ottawa, CA	N.A.	Christensen and Cook, 1978

*N.A. Not applicable/not available*.

*NL, The Netherlands; USA, United States of America; CA, Canada*.

### Soil collection and storage

The non-suppressive (conducive) soil to *R. solani* was collected from a pear orchard located in Zwaagdijk, The Netherlands (52°41′53.549″ N, 5°6′58.643″ E) in June 2012 at a depth of 10–40 cm. The soil, classified as clay soil with loam texture (29.9% of the particles are >50 μm, 26.4% of the particles are < 2 μm), was air-dried, sieved (0.5 cm mesh) to remove plant/root material and stored at 8°C until use for the *in vivo* activity test of *Lysobacter* spp. against *R. solani* on cauliflower.

### Genetic and phenotypic characterization of the *Lysobacter* strains

#### BOX-PCR

To determine the genetic variation among *Lysobacter* strains, the repetitive elements in their genome were analyzed by BOX-PCR according to Rademaker et al. (2004). Amplification reactions were conducted in 25 μl volume composed of 1 μl BOX-A1R primer (10 μM), 1.25 μl dNTPs (25 mM each), 0.4 μl BSA (10 mg/ml), 2.5 μl 100% DMSO, 5 μl 5x Gitschier buffer, 0.4 μl Taq polymerase (5U/μl SuperTaq), and 14.45 μl miliQ water. DNA was added by a toothpick inoculation of bacterial cells in the reaction mix. The reaction volume was heated to 95°C for 2 min, followed by 30 cycles of 3 s at 94°C, 92°C for 30 s, 50°C for 1 min, and 65°C for 8 min. The PCR reaction was finished with an 8 min incubation at 65°C for and then kept at 8°C. Five microliter of the PCR product was loaded on an 1.5% (w/v) agarose gel and ran overnight at 40V.

#### Phylogenetic analyses

For each *Lysobacter* strain, the sequences of the 16S ribosomal RNA gene, the gene encoding a recombination/repair protein (*recN*) and the gene encoding the subunit C of the excinuclease ABC (*uvrC*) were amplified using primers described in Table 2. The markers *recN* and *uvrC* were chosen based on Zeigler (2003) who showed that these candidate genes will provide high fidelity for species prediction, and the 16S rRNA gene was included because of its broad use in taxonomic studies. Amplification reactions were conducted in 25 μl volume composed of 1 μl each of forward and reverse primer (10 μM), 1 μl dNTPs (5 mM each), 1.5 μl MgCl_2_ (25 mM), 5 μl 5x GoTaq Flexibuffer, 0.125 μl GoTaq polymerase (5 U/μl), and 15.375 μl miliQ water. DNA was added by a toothpick inoculation of bacterial cells in the reaction mix. The reaction volume was heated to 95°C for 3 min, followed by 35 cycles of: 1 min at 95°C, 58°C for 1 min, 72°C for 1.4 min (for 16S rRNA), 1 min at 95°C, 57.2°C for 1 min, 72°C for 1.2 min (for *recN*), and 1 min at 95°C, 58°C for 1 min, 72°C for 2 min (for *uvrC*). The PCR reaction were finished with an 5 min incubation at 72°C for and then kept at 12°C. Five microliter of the PCR product were visualized on an 1.5% (w/v) agarose and PCR products were sequenced by Macrogen Inc. (Amsterdam, The Netherlands). Phylogenetic trees were constructed with the three markers independently or concatenated using ClustalW alignments (Thompson et al., 1994) and neighbor joining tree constructions using the Tamura 3 parameter model and discrete Gamma distribution in MEGA6 (Tamura et al., 2013).

**Table 2:** **Primer sets used for phylogenetic analysis**.

**Gene target**	**Primer**	**Oligonucleotides sequence (5^′^ → 3^′^)**
16S rRNA	Forward	AGAGTTTGATCCTGGCTCAG
16S rRNA	Reverse	ACGGGCGGTGTGTACA
*recN*	Forward	CTCAAGCAATTCGCCGTC
*recN*	Reverse	CACCTGCACCGCGCTCTG
*uvrC*	Forward	CGGCAAGGCCTTCGTCAAGC
*uvrC*	Reverse	CGTGCAAGGCGGCGTAGAT

The sequences obtained during this study are deposited in NCBI GenBank under accession numbers KT851449 to KT851466 for *uvrC*, KT851467 to KT851484 for 16S rRNA and KT851485 to KT851502 for *recN*.

#### Swarming ability

Motility of the *Lysobacter* strains was assessed on soft standard succinate medium (SSM) as described in De Bruijn and Raaijmakers (2009). In brief, 5 μl of *Lysobacter* suspensions was spot-inoculated in the center of SSM agar Petri dishes [(32.8 mM K_2_HPO_4_, 22 mM KH_2_PO_4_, 7.6 mM (NH_4_)_2_SO_4_, 0.8 mM MgSO_4_, 34 mM succinic acid (w/v)), adjusted pH to 7 and 0.6% agar (w/v)]. Petri dishes were incubated for 2–12 days at 25°C.

#### Enzymatic activity

Chitinase, glucanase, and protease activity of the *Lysobacter* strains were tested as described in De Bruijn et al. (in press). In brief, 2–5 μl of *Lysobacter* suspensions (of stationary phase of growth) was spot-inoculated in the center of different media containing 1.5–2% agar. For chitinase activity, R2A (Oxoid) and 1/10th strength TSB agar Petri dishes were used containing 0.2% colloidal chitin prepared from crab shell chitin (Sigma) and Petri dishes were incubated for 3–7 days at 25°C. For glucanase activity, R2A medium containing 0.5% laminarin was used and Petri dishes were incubated for 3 days at 25°C. The colonies were removed by washing with water and the medium was stained with 1% congo red. After destaining, coloration of the medium was determined. For protease activity, bacteria were inoculated on 15 g/l skimmed milk powder, 4 g/l blood agar base and 0.5 g/l yeast extract and Petri dishes were incubated for 3–7 days at 25°C.

#### In *Vitro* antagonistic activity

*Lysobacter* strains (Table 1) were grown in 5 ml TSB for 2 days at 25°C on a rotary shaker at 200 rpm. Suspensions were washed once by centrifugation at 3800 × g for 5 min and 10x concentrated in 0.9% NaCl.

To test activity against bacterial pathogens, R2A, 1/5th potato dextrose agar (PDA, Oxoid) and Luria-Bertani (LB, Difco) agar Petri dishes were prepared with an overlay of 1% water agar cooled down to 50°C to which washed cells of a culture of the bacterial pathogens (Table S1) were added. Subsequently, 2–5 μl of the *Lysobacter* cell suspensions (of stationary phase of growth) was spot-inoculated on the medium. Petri dishes were incubated for 3–7 days at 25°C and clearing zones surrounding the colonies were monitored.

To test inhibition of mycelial growth, oomycetes, and fungal strains (Table S1) were grown on PDA at 25°C. Four 5 μl of the *Lysobacter* suspensions were spot-inoculated at the edges of Petri dishes containing 20 ml of R2A, 1/5th PDA or PDA and a fresh 5 mm agar plug with actively grown mycelium was placed in the middle of the Petri dish.

To test antagonism against fungal spores, fungi (Table S1) were grown on PDA until sporulation. To enhance spore production, *Cercospora* and *Stemphylium* were grown on vegetable juice agar Petri dishes [(vegetable juice (V8) solified with 1.5% agar)] (Beckman and Payne, 1983; Rossi et al., 2005).

Under 16 h photoperiod, and to enhance spore collection from *Verticillium* and *Aspergillus*, the spores of those two fungi were scratched from the mycelium and streaked on fresh PDA Petri dishes. Fungal spores were collected as described in Trifonova et al. (2008) with slight modifications. In brief, spores were released from the mycelium by adding 10 ml of 0.9% NaCl and scratching the surface with a sterile spatula, collected, 10-fold diluted and added to the culture media (PDA, 1/5th PDA and R2A) of 48–55°C to a final concentration of 5% (v/v). Four 5 μl of the *Lysobacter* suspensions were spot-inoculated at the edges of Petri dishes containing 20 ml of medium with spores.

For each assay, three replicates per media were used. Petri dishes without *Lysobacter* were used as controls. All Petri dishes were incubated at 25°C for 1 week and subsequent inhibitory halo formation was monitored.

### *In Vivo* activity of *Lysobacter* spp. against *Rhizoctonia solani*

Spontaneous rifampicin-resistant mutants of the *Lysobacter* strains were verified by BOX-PCR. These mutants exhibited chitinase activity to the same extent as their parental strains. The rifampicin-resistant mutants were grown in 10 ml of TSB supplemented with 50 μg/ml rifampicin for 2 days at 25°C on a rotary shaker at 200 rpm. Cultures were centrifuged, washed 3 times and resuspended in 0.9% NaCl. Cell suspensions were mixed in a potting soil:river sand (1:9, w/w) mixture at an initial density of 10^7^ cells/g soil and approximately 20% hydration. Rectangle shape trays (19.5 × 6 × 3.5 cm) were filled with 250 g of the potting soil:sand mixture (eight replicates per treatment) and 16 sugar beet seeds coated with thiram, hymexazol, and poncho-beta were sown in a row, 1 cm apart. Non-inoculated soil was used as a control. Trays were placed in boxes with transparent lids in a growth chamber at 24°C with a 16 h photoperiod. After 5 days, seeds germinated and a single fresh 1/5th PDA agar plug (5 mm) grown with *R. solani* AG2-2 IIIB was placed touching the first seedling, with the mycelial side toward the plant. Spread of *R. solani* was scored at regular intervals during 2 weeks by scoring the number of diseased plants as well as the distance between the inoculum and the most distal plant suffering from damping-off. In addition, the area under the disease progress curve (AUDPC) was calculated to determine the disease dispersal over time as:

$$\left(Ak=\sum_{i\;=\;1}^{Ni-1}\frac{\left(y_{i}+y_{i+1}\right)}{2}\left(t_{i+1}-t_{i}\right)\right)$$

where *t*_*i*_ are the time points in a sequence (days) and y_*i*_ are measures of the disease dispersal (cm). Therefore, *y*(0) is defined as the initial infection at *t* = 0 and *A*(tx) is the AUDPC (total accumulated diseased dispersal until *t* = *t*_*x*_).

From each tray, the rhizospheres of two healthy sugar beet plants that were the closest to the last infected one were collected. Two replicates were pooled together in 4 ml 0.9% NaCl, vortexed for 1 min, sonicated for 1 min and vortexed for 15 s. Fifty microliter of a 10-, 100-, and 1000-fold dilution were plated on selective medium, R2A supplemented with 50 μg/ml rifampicin, 200 μg/ml ampicillin, 25 μg/ml kanamycin, and 100 μg/ml delvocid. Petri dishes were incubated at 25°C for 1 week. Colony forming units (CFU) were counted and CFU/g rhizosphere was calculated. The *in vivo* assay and the rhizosphere colonization test were done twice.

A similar experiment was performed in cauliflower using the same set up as described above with slight differences. Bacterial strains were grown in 10 ml of LB broth supplemented with 50 μg/ml rifampicin at 25°C for 3 days. The selected *Lysobacter* strains for this assay were L08, L14, L15, L19 and L29. Bacterial strains were inoculated in Zwaagdijk conducive soil at an initial density of 10^5^ and 10^7^ cells/g soil. Sowing, *R. solani* AG2-1/21 inoculation, growth of the plants, disease scoring, and AUDPC calculation was done as described above. The experiment was repeated twice, once with rifampicin resistant *Lysobacter* and once with non-rifampicin resistant *Lysobacter*. Statistically significant differences were determined by One-way ANOVA and *post-hoc* Dunnet's analysis (*P* < 0.05) performed in SPSS 22.0.

### *In Vitro* plant growth promotion assay

#### Seed preparation

Prior to surface sterilization, naked sugar beet (*Beta vulgaris*) seeds were soaked in 0.03 N HCl for 6 h under rotation, washed with sterile milliQ water and air-dried to enhance seed germination (Habib, 2010). Surface sterilization of sugar beet, cabbage (*Brassica oleracea*), and onion (*Allium cepa*) seeds was performed by washing the seeds in 2% sodium hypochlorite for 5 min and rinsing them with sterile milliQ water. Seeds were placed on Whatman filter paper moistened with 3 ml sterile milliQ water and pre-germinated at 25°C for 2–3 days. *Arabidopsis thaliana* (Columbia 0) seeds were sterilized in an exicator with 50 ml of commercial bleach (10% v/v) + 3% of concentrated HCl for 4 h, placed in wet Whatman filter paper and incubated at 4°C in darkness for 3 days.

#### Seed inoculation

Two day-old pre-germinated sugar beet seeds were soaked in 3 ml of *Lysobacter* suspensions of 10^9^ cells/ml for 30 min. Subsequently, sugar beet seeds (six seeds per container) were placed in cylinder shaped plastic containers (9 cm diameter, 8 cm height) with transparent lids containing 150 ml of 0.5 × Murashige and Skoog (MS) medium (including vitamins), and incubated in a growth chamber at 24°C with a 16 h photoperiod. Fresh and dry weight of shoots and roots were determined after 2 weeks. The experiment was done twice, with three replicates per treatment.

#### Root tip inoculation

Two days-old pre-germinated sugar beet seeds were placed in square Petri dishes (10 × 10 × 2 cm) containing 50 ml of 0.5 × MS medium (four seeds/Petri dish). Petri dishes were incubated in vertical position in a growth chamber at 24°C with a 16 h photoperiod until the roots were approximately 1 cm long and 2 μl of the *Lysobacter* suspensions of 10^9^ cells/ml were, spotted onto each root tip and incubated for 1 week. Fresh and dry weight of shoots and roots was determined. The experiment was done once, with three replicates per treatment.

#### Volatile assay

Two days-old pre-germinated seeds of sugar beet, cauliflower and onion were placed in containers as described above containing either 150 ml of 0.5 × MS medium or 150 g of a sterile mixture of potting soil:sand (1:9) with 20% humidity. A small Petri dish (35 mm diameter), containing 4 ml of R2A medium was placed in the middle of the container, and the *Lysobacter* strains were inoculated into the small Petri dishes at a density of 10^7^ cells/Petri dish. Containers were incubated in a growth chamber at 24°C with a 16 h photoperiod for 2 weeks and fresh and dry weight of shoots and roots as well as leaf area were determined. The experiment was performed three times for sugar beet, once for cauliflower and once for onion, with five replicates per treatment. For the volatile assay in *A. thaliana, L. antibioticus* L08, *L. capsici* L14, *L. gummosus* L15, and *Pseudomonas fluorescens* SBW25 [known by its ability in promoting plant growth in *A. thaliana* when growing on King's B (KB) agar medium and used as a positive control (J. M. Raaijmakers, personal communication)] were used. Each bacterial strain was pre-cultured in LB broth for 2 days at 25°C, and then washed three times with 10 mM MgSO_4_. A 10 μl drop of a bacterial suspension of 10^9^ cells/ml was spotted in the small Petri dish (35 mm diameter) containing 4 ml of R2A, LB or KB agar medium and Petri dishes were incubated for 1 day at 25°C. Small Petri dishes were placed into big Petri dishes (150 mm diameter) containing 50 ml of 0.5 × MS medium and five 3-days-old pre-germinated seeds were sown per Petri dish. Petri dishes with medium but without bacteria were included as controls. Petri dishes were incubated in vertical position in a growth chamber at 21°C with a 16 h photoperiod for 21 days. After that period, fresh and dry weight of shoots and roots were determined. The experiment was repeated once with five replicates/treatment.

### Seed colonization ability

Naked sugar beet seeds were surface sterilized as described above and soaked in 3 ml of bacterial suspensions containing 10^9^ cells/ml for 30 min as described above for the seed inoculation assay (22 seeds/bacterial treatment). Six seeds from each bacterial suspension were placed in 4 ml 0.9% NaCl, vortexed 1 min, sonicated 1 min, and vortexed 15 s. Fifty microliter of both undiluted suspensions and 10, 100, 1000, and 10000x time dilutions were plated on R2A agar dishes and incubated at 25°C for 1 week. The remaining seeds were sown in squared Petri dishes containing 50 ml of 0.5 MS (four seeds/Petri dish, four replicates per treatment) and incubated as described above for the root tip inoculation assay. After 1 week, the roots of the seedlings from each Petri dish were excised and placed in 4 ml of 0.9% NaCl, vortexed 1 min, sonicated 1 min, and vortexed 15 s. Fifty microliter of both undiluted suspensions and 10, 100, 1000, and 10000x fold dilution were plated on R2A agar dishes, incubated at 25°C for 1 week and the amount of colony forming units (CFU) per seed and per root were determined by colony counting.

## Results

### Genetic and phenotypic characterization of the *Lysobacter* strains

**Figure 1:**
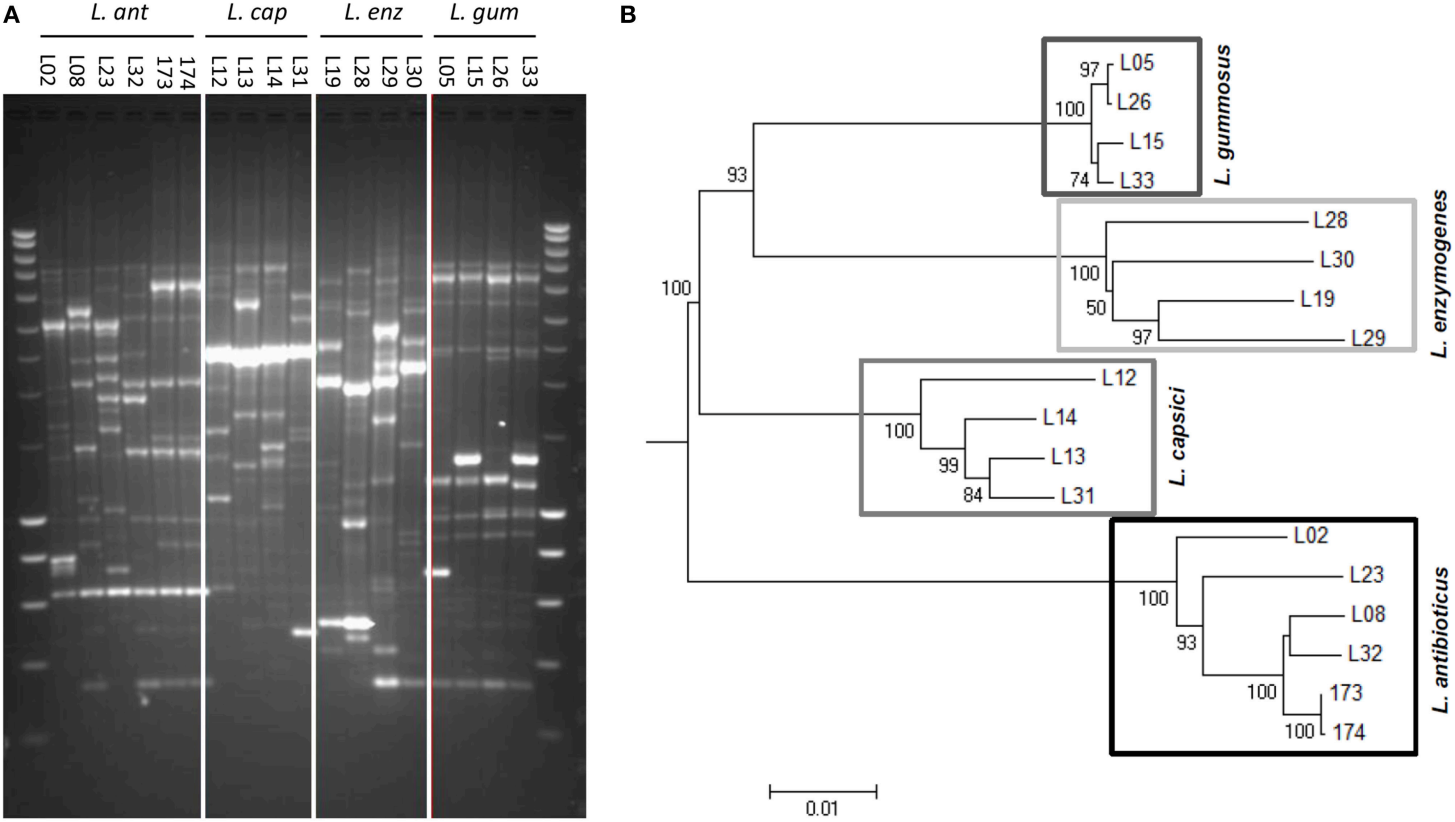
**Genetic diversity of 18 selected *Lysobacter* strains belonging to four different species**. **(A)** Genetic profiling by BOX-PCR. Lanes on complete left and right shows Smartladder (Eurogentec) marker. **(B)** Phylogenetic tree of the *Lysobacter* strains based on the concatenated sequences of the 16S ribosomal RNA gene (16S rRNA), a gene encoding a recombination/repair protein (*recN*) and a gene encoding the subunit C of the excinuclease ABC (*uvrC*). The evolutionary relationship of the *Lysobacter* strains was inferred by alignment with ClustalW and neighbor-joining tree construction. The numbers at the nodes indicate the level of bootstrap support of 50 or higher, based on neighbor-joining analysis of 1000 resampled data sets. The bar indicates the relative number of substitutions per site.

BOX-PCR profiling of the 18 *Lysobacter* strains revealed a high genetic diversity among the different *Lysobacter* species and between strains of a given species (Figure 1A). *L. gummosus* strains showed the lowest intraspecific diversity whereas *L. enzymogenes* strains showed the highest diversity. Based on 16S rRNA sequences, the most phylogenetically distant species was *L. enzymogenes* (Figure S1A). When using either *recN* or *uvrC* or the three molecular markers together, however, *L. antibioticus* was the most distant of the four species (Figure 1B and Figures S1B,C).

**Figure 2:**
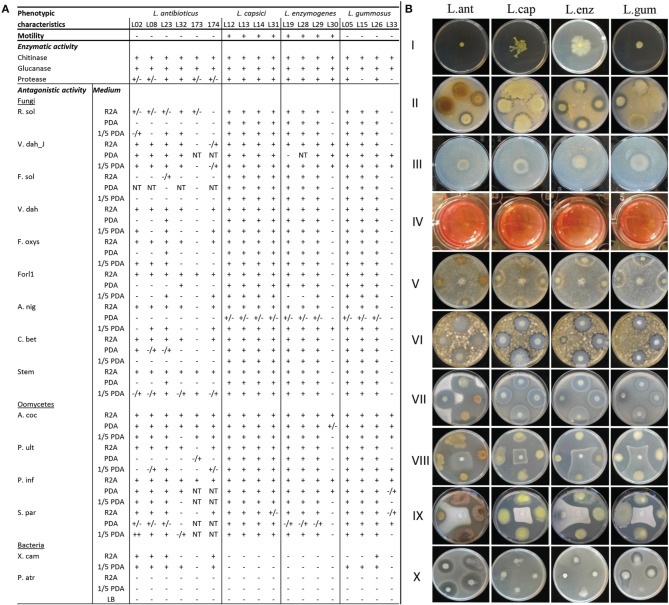
**Phenotypic characterization of the *Lysobacter* strains, including (A) motility, protease, chitinase and glucanase activities, and antagonistic activity against pathogenic fungi, oomycetes and bacteria**. + indicates activity; − indicates no activity; ± indicates antagonistic activity observed after 2–3 days of incubation, but the activity disappeared upon longer incubation. For the enzymatic activity, the ± indicates weak activity; NT indicates not tested. **(B)** Pictures of phenotypic characterization of *L. antibioticus* (L. ant), *L. capsici* (L. cap), *L. enzymogenes* (L. enz), and *L. gummosus* (L. gum) for I: motility on SSM medium; II: chitinase activity; III: glucanase activity, positive glucanase activity is given by the change from red to orange color (not shown); IV: protease activity; and *in vitro* antagonistic activity on R2A (except when otherwise indicated) against V: *R. solani*; VI: *Cercospora beticola*; VII: *Verticillium dahliae*; VIII: *Pythium ultimum*; IX: *Aphanomyces cochlioides* on PDA and X: *Xanthomonas campestris* pv. *campestris* on 1/5th PDA.

The *Lysobacter* strains did not show any motility after 4 days of incubation on soft SSM agar medium. After 12 days of incubation, however, *L. capsici* (L12, L13, L14, and L31) and *L. enzymogenes* (L19, L28, L29, L30) did spread from the point of inoculation, most likely due to gliding motility (Figure 2). All *Lysobacter* strains used in this study showed extracellular chitinase and glucanase activities (Figure 2). Most strains presented proteolytic activity except for two *L. gummosus* and four *L. antibioticus* strains (Figure 2). Variation in these three enzymatic activities among strains belonging to the same species was observed, especially for the *L. antibioticus* strains.

The antimicrobial activity of the *Lysobacter* strains (Table 1) was tested on different media. Almost all *Lysobacter* strains showed a strong antagonistic activity against all pathogens tested (Table S1), except against the plant pathogenic bacterium *Pectobacterium atrosepticum*. The magnitude of the antagonistic activity of *Lysobacter* was media-dependent, with the strongest activity on R2A medium and the weakest activity on PDA medium (Figure 2). *L. capsici* was the most consistent species in terms of antagonistic activity, with all *L. capsici* strains showing activity on R2A against all pathogens tested except for *X. campestris* and *L. capsici* strain L31 against *S. parasitica* (Figure 2). On R2A, all *L. enzymogenes* and *L. gummosus* strains, with the exception of the type strains, showed activity against all pathogens tested. The type strain of *L. enzymogenes* did show activity against *V. dahliae* JR2, *A. cochlioides* and *P. infestans*, whereas the *L. gummosus* type strain had activity against all oomycetes tested except *P. ultimum* (Figure 2). *L. antibioticus* strains showed the highest variation in activity, with strain L23 having the broadest antimicrobial activity (Figure 2).

### *In Vivo* activity of *Lysobacter* spp. against *Rhizoctonia solani*

**Figure 3:**
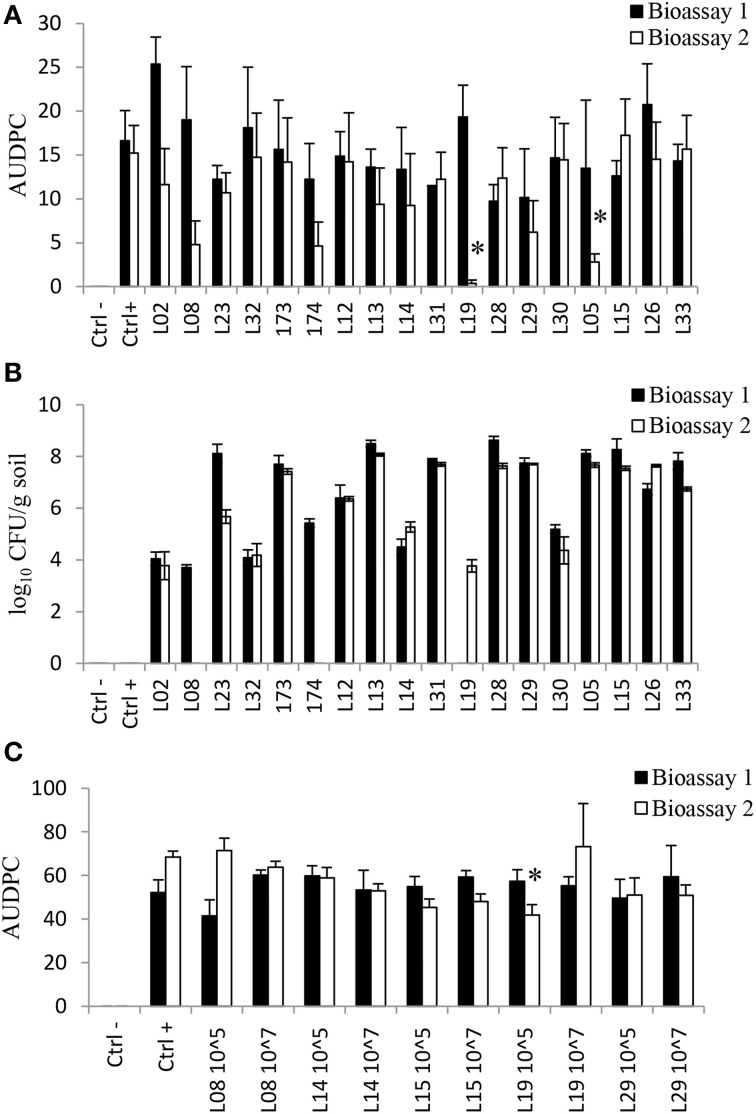
***In vivo Rhizoctonia* disease suppression and rhizosphere colonization ability by *Lysobacter* strains**. **(A)** Area under disease progress curve (AUDPC) of disease spread for sugar beet when *Lysobacter* strains were applied at an initial density of 10^7^ CFU/g into a mixture potting soil:sand (1:9); **(B)** Colonization of the rhizosphere of sugar beet by the *Lysobacter* strains when applied at an initial density of 10^7^ CFU/g into a mixture potting soil:sand (1:9). **(C)** AUDPC of disease spread for cauliflower when *Lysobacter* strains were applied into a conducive soil. 10^7 and 10^5 means an initial density of the inoculum at 10^7^ and 10^5^ cells/g soil, respectively; *L. antibioticus*: L02, L08, L23, L32, 173, 174; *L. capsici*: L12, L13, L14, L31; *L. enzymogenes*: L19, L28, L29, L30, and *L. gummosus*: L05, L15, L26, L33. For each of the two bioassays, an asterisk indicates a significant difference (*p* < 0.05) with the control treatment calculated by analysis of variance and Dunnet's *post-hoc* analysis.

The efficacy of the *Lysobacter* strains, several of which originate from *Rhizoctonia* suppressive soil, to control *Rhizoctonia* damping-off disease of sugar beet seedlings was tested in a sterilized (by autoclaving twice) sand-potting soil mixture and in a non-sterilized agricultural soil. Seed germination was not affected by the *Lysobacter* strains. In two bioassays, none of the strains was able to consistently suppress damping-off disease caused by *R. solani* after 2 weeks of plant growth (Figure 3A). For example, strains L19 and L05 significantly reduced damping-off disease of sugar beet in bioassay 2 but not in bioassay 1 (Figure 3A).

The results further showed that after an initial application of 10^7^ CFU/g soil, *Lysobacter* strains established densities in the rhizosphere of sugar beet ranging from 10^3^ to 10^8^ CFU/g (Figure 3B), with substantial variation between strains and between the two bioassays. In general, *L. gummosus* strains were better rhizosphere colonizers whereas *L. antibioticus* showed the highest variation among strains. *L. antibioticus* strains L08 and 174 were only detected in the sugar beet rhizosphere in bioassay 1. *L. antibioticus* L23 was detected at high densities (10^8^ CFU/g) in bioassay 1, but at 1000-fold lower densities in bioassay 2. *L. enzymogenes* L19 was only detected in bioassay 2 (Figure 3B).

The ability of *Lysobacter* to suppress *Rhizoctonia* damping-off disease of another host plant (cauliflower) was assessed for *Lysobacter* strains L08, L14, L15, L19, and L29 at two initial densities of 10^5^ and 10^7^ CFU/g of soil. Also for this crop, germination was not affected by the introduced bacterial strains and again no significant and consistent reduction in disease incidence was observed. When applied at 10^5^ CFU/g of soil, strain L19 significantly reduced disease incidence but only in bioassay 2 (Figure 3C). For bioassay 2, colonization of cauliflower rhizosphere by the *Lysobacter* strains was determined. The results showed that the densities recovered were lower (10^1^ to 10^3^) than initially applied except for *L. enzymogenes* L29 and *L. gummosus* L15 when applied at 10^7^ CFU/g soil (Figure S2). After an initial application of 10^5^ cells/g soil, only *L. gummosus* L15 and *L enzymogenes* L19 and L29 were detected in the rhizosphere of cauliflower.

### Plant growth promotion

**Figure 4:**
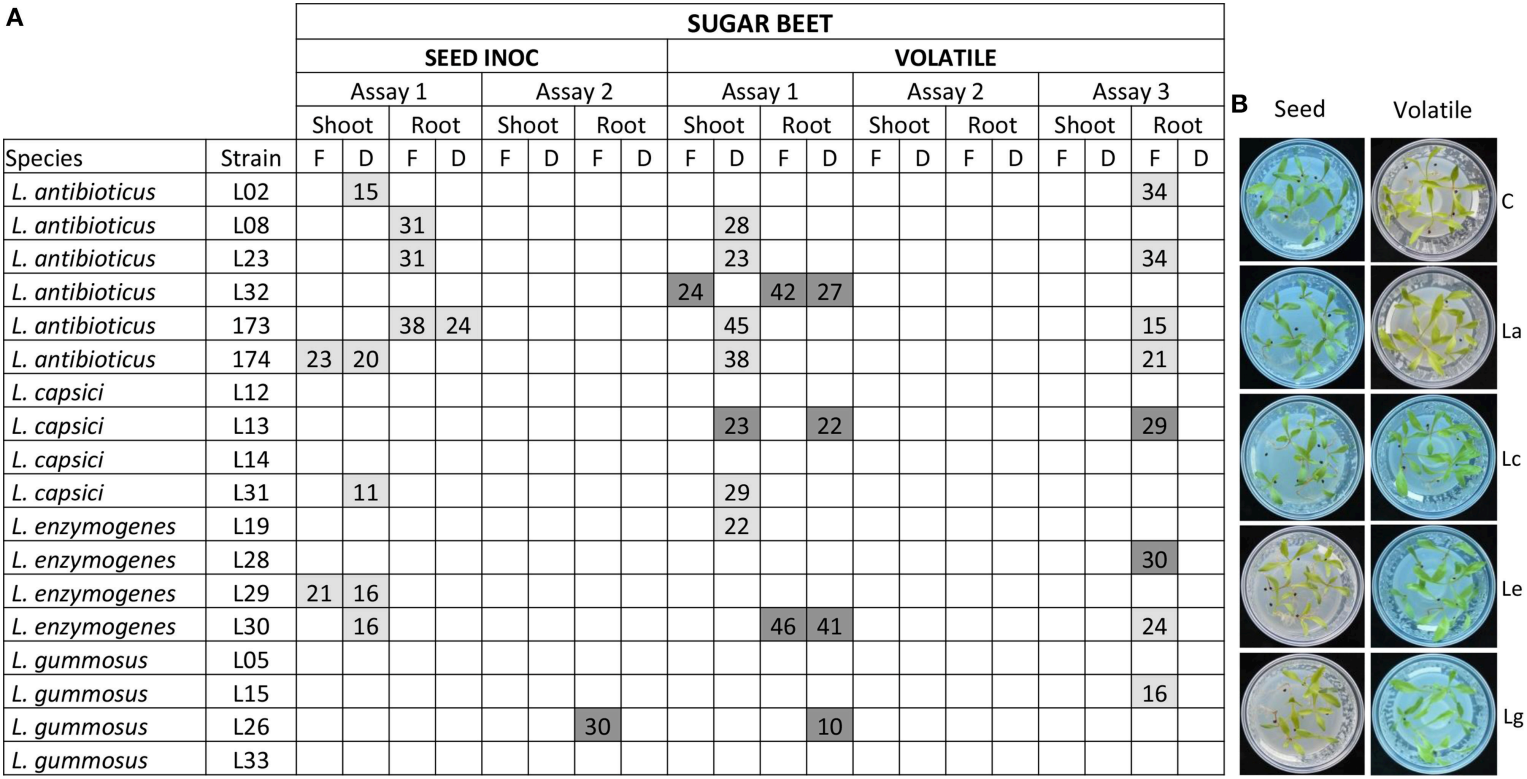
**Sugar beet plant growth promotion by *Lysobacter* strains**. **(A)** Sugar beet seeds were grown on 0.5 MS medium and plant growth promotion was determined when *Lysobacter* strains were inoculated on seeds or by volatiles. Each assay was performed with three to five replicates. F indicates fresh weight; D indicates dry weight. Light gray boxes indicate a statistical significant negative effect in plant growth when compared to the control and dark gray boxes indicate a statistical significant positive effect. Values within the boxes, indicates the % of increase/decrease of plant weight compared to the control. **(B)** Pictures of the plant growth promotion assays. C, control; La: *L. antibioticus*; Lc: *L. capsici*; Le: *L. enzymogenes*; Lg: *L. gummosus*. Significant differences (*p* < 0.05) with the uninoculated control were calculated using analysis of variance and Dunnet's *post-hoc* analysis.

The ability of the *Lysobacter* strains to promote plant growth *in vitro* was tested for sugar beet, cauliflower, onion, and *A. thaliana*. For sugar beet, the 18 *Lysobacter* strains were applied to the seeds as well as to the root tips. For the first seed inoculation assay, almost all *L. antibioticus* strains negatively affected plant growth, decreasing plant biomass with 15–38% compared to the untreated control (Figure 4). One *L. capsici* and two *L. enzymogenes* strains negatively affected shoot biomass. In the second bioassay, no negative or positive effects on plant growth were observed for any of the strains (Figure 4), except for *L. gummosus* L26 which promoted root growth.

The ability of *Lysobacter* to colonize the surface of the seeds and the roots was determined for bioassay 2. Whilst bacteria were applied at an initial density of 10^8^ cells/seed, bacterial recovery from the seed after 30 min of incubation ranged from approximately 10^3^–10^4^ cells/seed, with even lower numbers for *L. antibioticus* L32 (10^2^ cells/seed; Table S2). After 1 week of plant growth, bacteria could not be detected on sugar beet roots (Table S2). Hence, *Lysobacter* appears to be a poor root colonizer under these experimental conditions.

In the root tip inoculation assay, positive effects (ranging from 17 to 28% biomass increase) were observed for dry weight of shoots by two *L. antibioticus*, two *L. capsici*, and one *L. enzymogenes* strains (Figure S3). One *L. antibioticus* and one *L. gummosus* strain increased fresh (33%) and dry (38%) root biomass respectively (Figure S3).

To determine if *Lysobacter* emits volatile compounds that promote plant growth, assays were conducted in a split Petri dish where *Lysobacter* was physically separated from sugar beet seedlings. A high variation in plant phenotypes was observed between assays. For example, *L. antibioticus* L32 increased shoot biomass with 24% and root biomass with 42% only in the first assay. *L. enzymogenes* L30 increased root biomass in the first assay whereas in the third assay it showed a negative effect on plant growth (Figure 4). The volatile assays were repeated in sterile potting soil:sand mixture with sugar beet, cauliflower, and onion. Also in these assays, no significant and consistent results were obtained for the *Lysobacter* strains tested (data not shown). In addition, plant growth promotion was also determined by measurement of the leaf surface and no positive or negative effects of the *Lysobacter* strains were observed (data not shown).

*L. antibioticus* strain L08, *L. capsici* L14, *L. gummosus* L15 were also tested for volatile-mediated growth promotion of *A. thaliana* on different media. The positive control *P. fluorescens* SBW25 significantly increased shoot and root biomass (Figure S4). However, none of the *Lysobacter* strains tested showed a plant growth promoting effect on *A. thaliana*. Furthermore, when growing on LB medium, all the three *Lysobacter* as well as *P. fluorescens* SBW25 showed a notable adverse effect on plant growth (Figure S4).

## Discussion

The genus *Lysobacter* is receiving substantial ecological and biotechnological interest as producers of different exoenzymes and antibiotics (Pidot et al., 2014). During the last years, several *Lysobacter* species have been isolated from Dutch soils suppressive to the fungal root pathogen *R. solani* (Postma et al., 2008, 2010b). Here, we showed that 18 *Lysobacter* strains from *Rhizoctonia* suppressive soils showed a high genetic diversity. In a recent study, comparative genomics of seven *Lysobacter* strains (five of which are included in this study) belonging to four *Lysobacter* species showed only 55% overlap in genome content (De Bruijn et al., in press). A high genetic diversity can confer an advantage under adverse environmental conditions as some members may exhibit phenotypes that allow them to survive and proliferate (Foster, 2005). Genome analysis also revealed the lack of flagellar genes (De Bruijn et al., in press), which supports our findings that none of the *Lysobacter* strains tested were motile on soft agar. Nonetheless, some dispersal was observed for *L. capsici* and *L. enzymogenes* after 12 days of incubation, most likely due to gliding motility as described previously for other *Lysobacter* species (Sullivan et al., 2003; Hayward et al., 2010).

*Lysobacter* is known to produce a variety of bioactive compounds, including enzymes and antimicrobial compounds. Hence, they were pointed out as an untapped source of new bioactive products (Xie et al., 2012; Pidot et al., 2014). Our results showed that the *Lysobacter* strains possess chitinase and glucanase activity, confirming and extending previous research (Zhang and Yuen, 2000; Zhang et al., 2001; Palumbo et al., 2005; De Bruijn et al., in press). Protease activity was observed for all strains belonging to *L. capsici* and *L. enzymogenes*, whereas only two out of four strains from *L. gummosus* and two out of six from *L. antibioticus* showed this activity. Chitinase, glucanase and protease activities may contribute to antimicrobial activity, since chitin, α- and β-glucans and glycoproteins are the major components of the cell walls of fungi (Barreto-Bergter and Figueiredo, 2014).

Most of the *Lysobacter* strains effectively inhibited the growth of oomycetes and fungi; only *L. antibioticus* and *L. gummosus* strains showed antibacterial activity. Differences in activity were observed between *Lysobacter* species and between strains of a given species, suggesting that the genus *Lysobacter* indeed may have a large reservoir of putative novel bioactive compounds. The *in vitro* antagonistic activity was media-dependent, showing stronger activity on poor medium, confirming and extending results obtained previously for the activity of *L. enzymogenes* 3.1T8 against *Pythium aphanidermatum* (Folman et al., 2004).

Due to their broad spectrum activity, *Lysobacter* members have been proposed as promising candidates for biological control of plant diseases (Hayward et al., 2010). However, none of the *Lysobacter* strains used in this study were able to consistently reduce *R. solani* infection on sugar beet and cauliflower. These results differ from those in previous studies where several *Lysobacter* strains significantly controlled plant pathogens, including *P. aphanidermatum* on cucumber (Folman et al., 2004; Postma et al., 2009), *Bipolaris sorokiniana* on tall fescue (Kilic-Ekici and Yuen, 2003), *Uromyces appendiculatus* on bean (Yuen et al., 2001), *Xanthomonas oryzae* pv. *oryzae* on rice (Ji et al., 2008), *Phytophthora capsici* on pepper (Ko et al., 2009), *Plasmopara viticola* on grapevine (Puopolo et al., 2014), *Aphanomyces cochlioides* in sugar beet and spinach (Islam et al., 2005) and *F. oxysporum* f. sp. *radicis-lycopersici* on tomato (Puopolo et al., 2010). Furthermore, *L. capsici* YS1215 was reported to have nematicidal activity, reducing root-knot caused by *Meloidogyne incognita* by inhibiting egg hatching (Lee et al., 2014).

Most of the *Lysobacter* strains tested here poorly colonized the rhizosphere of sugar beet and cauliflower. Given the importance of root colonization for biocontrol (Bull et al., 1991; Johnson, 1994; Raaijmakers et al., 1995), this suggests that the inconsistency in disease control by the *Lysobacter* strains may be due to their lack of competitiveness in the rhizosphere of sugar beet and cauliflower. The rhizosphere differs from the bulk soil by the presence of plant root exudates that create an environment rich in nutrients. Chemotaxis and active motility toward root exudates represent the first steps in rhizosphere colonization (Benizri et al., 2001; De Weert and Bloemberg, 2006). This motility may be active, through flagellar movements, or passive, through percolating water or vectors. None of the 18 *Lysobacter* strains possess flagella, what limits the capacity of the strains to effectively compete against flagellated soil bacteria for a niche in the rhizosphere. The adherence to root tissues through biofilm formation is the next step in rhizosphere colonization (Benizri et al., 2001; Ramey et al., 2004; Danhorn and Fuqua, 2007). Several traits are involved in biofilm formation including cell wall structures and extracellular polysaccharide production (Lugtenberg et al., 2001). Biofilm production *in vitro* has been described for *L. capsici* AZ78 and appeared medium specific, (Puopolo et al., 2014). Biofilm formation was observed for *Lysobacter* sp. strain SB-K88 on roots of sugar beet (Islam et al., 2005). Biofilm formation *in situ* was not tested for our 18 *Lysobacter* strains and will be subject of future studies. The root exudate composition is plant specific (Mandimba et al., 1986) and the ability to assimilate specific amino acids, vitamin B1, carbohydrates, organic acids as well as pH tolerance and competition for limiting resources also determine the rhizosphere competence (Dekkers et al., 1999; Benizri et al., 2001; Lugtenberg and Kamilova, 2009; Ghirardi et al., 2012). In the rhizosphere there is often a limitation for soluble iron, commonly used as a cofactor in enzymes that are involved in pathways that are essential for microbial growth. Therefore, the ability to produce siderophores (small high-affinity iron chelating compounds) confers a competitive advantage. The role of competition for iron by siderophore production of *Lysobacter* sp. seems species or strain specific and not all strains, including several strains used in this study, possess iron-chelating capacity (Puopolo et al., 2010; Ko et al., 2011; De Bruijn et al., in press).

The soil type may also influence rhizosphere colonization and biocontrol activity. For example, the colonization of *Pseudomonas* sp. strain ITRI53 and *Pantoea* sp. strain BTRH79 of Italian ryegrass was higher in loamy soils compared with sandy soils (Afzal et al., 2011). The agricultural soil used in this study is a clay soil with loam texture. Several of our *Lysobacter* strains were isolated from this agricultural soil and we expected that those conditions would provide a “home-field advantage” for rhizosphere colonization of sugar beet and cauliflower. In a potting soil:sand mixture, we observed higher rhizosphere population densities on sugar beet seedlings as compared to the agricultural soil, with densities higher than the minimal dose of 10^5^ CFU/g soil reported for other biocontrol strains (Xu and Gross, 1986; Leeman et al., 1995; Raaijmakers et al., 1995). Despite these densities, no significant and/or consistent biocontrol activity was observed for any of the *Lysobacter* strains tested.

Several biocontrol agents not only suppress disease but also promote plant growth (Johansson et al., 2003). None of the *Lysobacter* strains tested in this study, however, were able to significantly and consistently promote growth of 4 different crops when applied to seeds or root tips or when applied physically separated from the crop. Furthermore, volatiles produced by the *Lysobacter* strains when grown on LB medium even showed a negative effect on growth of *A. thaliana*. This may be due to the accumulation of toxic volatiles that are produced by *Lysobacter* spp. when growing in rich media. Weise et al. (2013) showed that *Serratia odorifera* inhibited the growth of *A. thaliana* plants due to the production of ammonia when grown on peptone-rich nutrient media. Iwata et al. (2010) reported that *Lysobacter* sp. E4 was able to fix nitrogen under free-living conditions and accumulated ammonia in the culture broth. Also hydrogen cyanide (HCN) produced by *Chromobacterium, Pseudomonas*, and *Serratia* have been shown to inhibit the growth of *A. thaliana* (Blom et al., 2011). More research needs to be conducted to determine if HCN or other toxic volatiles are produced by *Lysobacter*.

Overall, our results indicate that none of the 18 *Lysobacter* strains have the potential to control *Rhizoctonia* or promote plant growth of sugar beet and cauliflower, probably due to insufficient rhizosphere competence. However, the *Lysobacter* strains showed a high diversity in *in vitro* activity against 14 different pathogenic fungi, oomycetes and bacteria, suggesting that the genus *Lysobacter* constitutes an extensive source of (new) enzymes and antimicrobial compounds. Possibly *Lysobacter* needs to interact with a specific microbial community to become antagonistic to *Rhizoctonia* or to promote plant growth in natural environments. To better understand the potential contribution of *Lysobacter* species to the overall activities of the microbial communities responsible for soil suppressiveness against *R. solani*, in-depth metagenomic and metatranscriptomic analyses of the bacterial community compositions and functions will be needed to unravel the role of this genus in disease suppressiveness. Future work will include testing *Lysobacter* mixtures or mixtures with other bacterial genera abundant in soils suppressive to *R. solani*. Interactions of *Lysobacter* with other bacteria may stimulate the production of antimicrobial compounds as was shown recently for other bacterial genera (Tyc et al., 2014).

## Author contributions

All authors were involved in the design of the experiments. RG and IB performed *in vitro* and *in vivo* activity bioassays, BOX-PCR and phylogenetic analyses. RG performed plant growth promotion assays. All authors contributed to the writing of the manuscript and approved submission.

### Conflict of interest statement

The authors declare that the research was conducted in the absence of any commercial or financial relationships that could be construed as a potential conflict of interest.
