# Supplementary material for: Diversity and Activity of Lysobacter Species from Disease Suppressive Soils
Source: Front Microbiol. 2015 Nov 16;6:1243. doi: 10.3389/fmicb.2015.01243 (PMC4644931; doi:10.3389/fmicb.2015.01243)
Supplement: Supplementary file 1 [file DataSheet1.PDF]

Supplementary material

Diversity and activity of *Lysobacter* species from disease suppressive soils

Ruth Gómez Expósito[1,2], Joeke Postma[3], Jos M. Raaijmakers[1] and Irene de Bruijn[1*]

***Correspondence:** Dr. Irene de Bruijn: i.debruijn@nioo.knaw.nl

Contents:

Figure S1: Phylogenetic trees of the 18 *Lysobacter* strains based on 16S ribosomal RNA gene (16S rRNA), recombination/reparation protein (*recN*) and excinuclease ABC (*uvrC*).

Figure S2: Colonization of *Lysobacter* strains of the rhizospheres of cauliflower plants.

Figure S3. Sugar beet plant growth promotion by *Lysobacter* strains when inoculated on root tips.

Figure S4. *Arabidopsis thaliana* plant growth promotion by *Lysobacter* volatiles.

Table S1. Pathogens used in this study

Table S2. *In vitro* colonization of the *Lysobacter* strains of the seed and root surface of sugar beet seedlings.

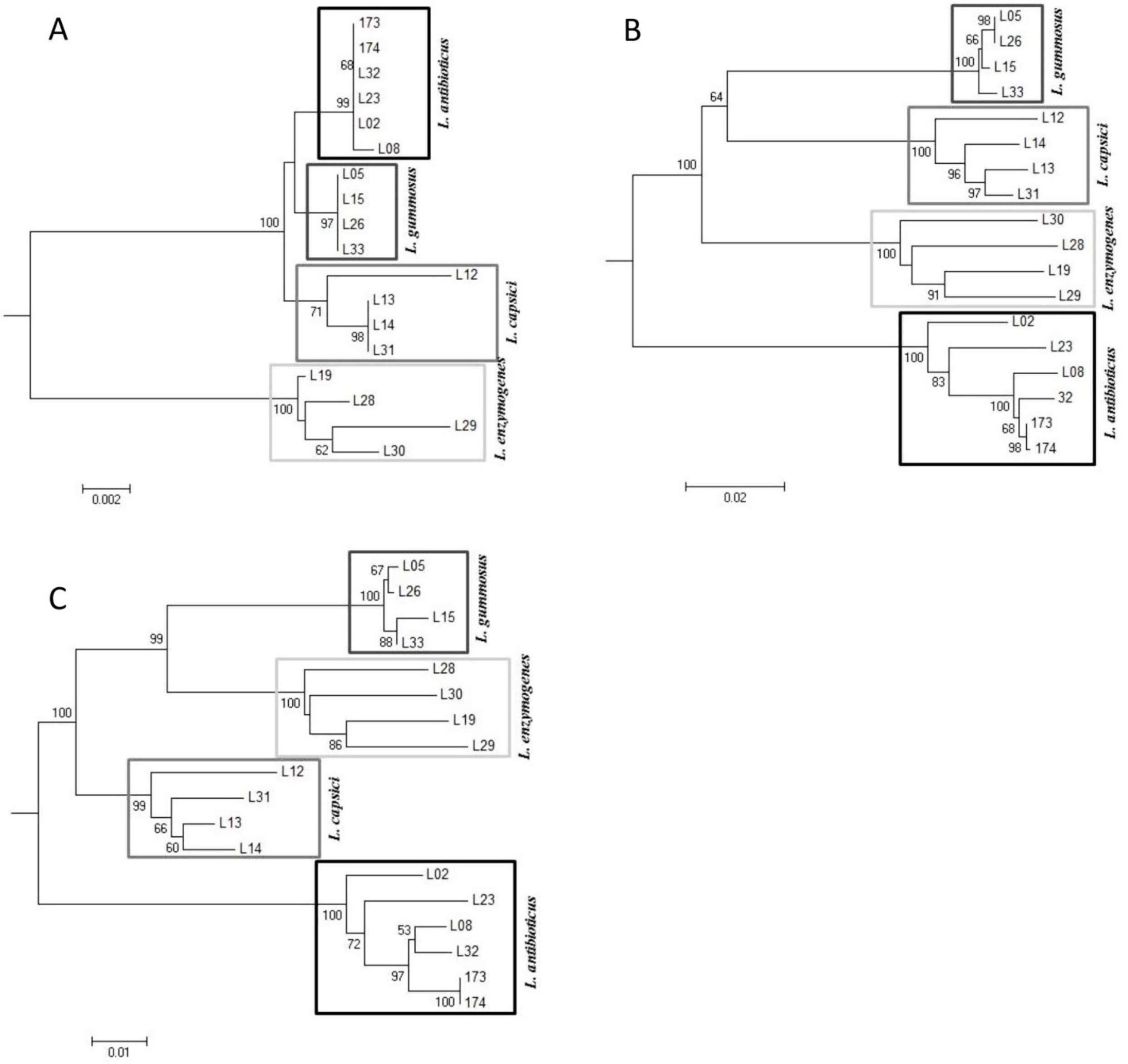

Figure S1. Phylogenetic trees of the 18 *Lysobacter* strains based on A) 16S ribosomal RNA gene (16S rRNA), B) recombination/reparation protein (*recN*) and C) excinuclease ABC (*uvrC*). The

evolutionary relationship of the *Lysobacter* strains was inferred by alignment with ClustalW and tree construction using the neighbor-joining method in MEGA6.

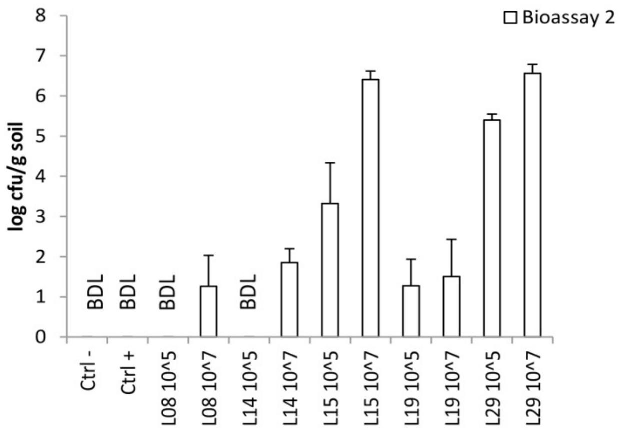

Figure S2. Colonization of *Lysobacter* strains of the rhizospheres of cauliflower plants. *L. antibioticus*: L08; *L.capsici*: L14; *L. gummosus*: L15 and *L. enzymogenes*: L19 and L29. 10^7 means an initial density of the inoculum at 10^7 CFU/g soil; 10^5 means an initial density of the inoculum at 10^5 cells/g soil. BDL means below detection limit (estimated in 232 CFU/g rhizosphere).

A

		SUGAR BEET			
		ROOT INOC			
		Assay 1			
		Shoot		Root	
Species	Strain	F	D	F	D
L. antibioticus	L02				
L. antibioticus	L08				
L. antibioticus	L23				
L. antibioticus	L32				
L. antibioticus	173		28		
L. antibioticus	174		27		38
L. capsici	L12		28		
L. capsici	L13		23		
L. capsici	L14				
L. capsici	L31				
L. enzymogenes	L19		17		
L. enzymogenes	L28				
L. enzymogenes	L29				
L. enzymogenes	L30				
L. gummosus	L05				
L. gummosus	L15				
L. gummosus	L26				
L. gummosus	L33			33	

B

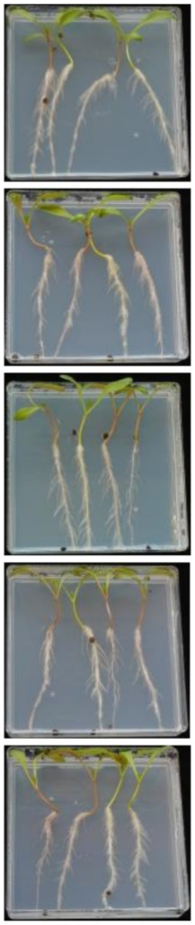

Figure S3. Sugar beet plant growth promotion by *Lysobacter* strains when inoculated on root tips. (A) Sugar beet seeds were grown on 0.5 MS medium and plant growth promotion was determined when *Lysobacter* strains were inoculated on the root tip. The assay was performed once with three replicates per treatment. F indicates fresh weight; D indicates dry weight. Light grey boxes indicate a statistical significant negative effect in plant growth when compared to the control and dark grey boxes indicate a statistical significant positive effect. Values within the boxes indicates the % of increase/decrease of plant weight compared to the control. **(B)** Pictures of the plant growth promotion assay. C: control; La: *L. antibioticus*; Lc: *L. capsici*; Le: *L. enzymogenes*; Lg: *L. gummosus*. Significant differences ($p<0.05$) with the uninoculated control were calculated using analysis of variance and Dunnet's post-hoc analysis.

A

			Arabidopsis thaliana			
			VOLATILE			
			Shoot		Root	
**Species**	**Strain**	**Medium**	F	D	F	D
Control	Control	NM				
Control	Control	R2A				
Control	Control	KB				
Control	Control	LB				
L. antibioticus	L08	R2A				
L. antibioticus	L08	KB				
L. antibioticus	L08	LB	95	88	91	88
L. capsici	L14	R2A				
L. capsici	L14	KB				
L. capsici	L14	LB	90	90	94	87
L. gummosus	L15	R2A				
L. gummosus	L15	KB				
L. gummosus	L15	LB	64		86	
P. fluorescens	SBW25	R2A				
P. fluorescens	SBW25	KB	177	308	147	176
P. fluorescens	SBW25	LB	91	92	74	

B

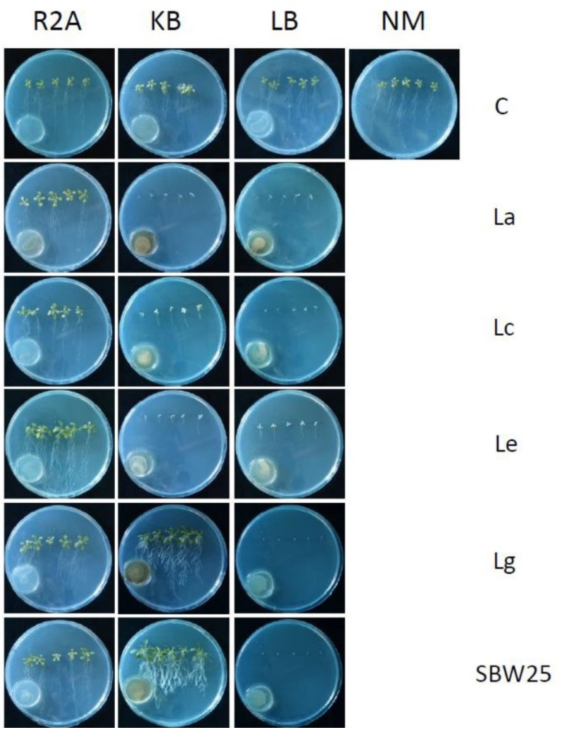

Figure S4. *Arabidopsis thaliana* plant growth promotion by *Lysobacter* volatiles. (A) Effect of the volatiles of three *Lysobacter* species and the positive control *Pseudomonas fluorescens* SBW25 when grown on different media: R2A, KB and LB. NM means no media added (control). The assay was performed once with 5 replicates. F indicates fresh weight; D indicates dry weight. Light grey boxes indicate a statistical significant negative effect in plant growth compared to the control and dark grey boxes indicate a statistical significant positive effect. Values within the boxes indicates the % of increase/decrease of plant weight compared to the control. **(B)** Pictures of the plant growth promotion assay. C: control; La: *L. antibioticus*; Lc: *L. capsici*; Le: *L. enzymogenes*; Lg: *L. gummosus*. Significant differences ($p<0.05$) with the uninoculated control were calculated using analysis of variance and Dunnet's post-hoc analysis.

Table S1. Pathogens used in this study

**Organism**	**ID code**	**Species**	**Strain**	**Propagule**	**Media used for growth/sporulation**
Fungus	R. sol	*Rhizoctonia solani*	AG2-2 III b	mycelium	PDA
Fungus	F. sol	*Fusarium solani*	F2	mycelium	PDA
Fungus	V. dah_J	*Verticillium dahliae*	JR2	mycelium	PDA
Fungus	F. oxys	*Fusarium oxysporum*	gN 07-047 d1	spores	PDA
Fungus	Forl1	*Fusarium oxysporum*	Forl1	spores	PDA
Fungus	V. dah	*Verticillium dahliae*	gN 10-188	spores	PDA
Fungus	A. nig	*Aspergillus niger*	N400	spores	PDA
Fungus	C. bet	*Cercospora beticola*	BV 1133 ga1	spores	V8
Fungus	Stem	*Stemphylium* sp.	BV 10-140 a1	spores	V8
Oomycete	A. coc	*Aphanomyces cochlioides*	HL B-22	mycelium	PDA
Oomycete	P. ult	*Pythium ultimum*	SB	mycelium	PDA
Oomycete	P. inf	*Phytophthora infestans*	88069	mycelium	PDA
Oomycete	S. par	*Saprolegnia parasitica*	CBS223.65	mycelium	PDA
Bacterium	X. cam	*Xanthomonas campestris* pv *campestris*	ZTO281	cells	LB
Bacterium	P. atr	*Pectobacterium atrosepticum*	SCR1	cells	LB

Table S2. *In vitro* colonization of the *Lysobacter* strains of the seed and root surface of sugar beet seedlings. Colonization densities of seeds are retrieved upon pooling 6 seeds and dilution plating. Colonization densities of roots are retrieved upon pooling 4 roots per plate and dilution plating; averages of 4 plates are indicated. BDL indicates below detection limit (estimated in $2*10^2$ CFU/root).

**Species**	**Strain**	**CFU/seed ($x10^4$)**		**CFU/root**
	Control	BDL		BDL
L. antibioticus	L02	40.0	± 3.3	BDL
L. antibioticus	L08	37.0	± 9.5	BDL
L. antibioticus	L23	25.0	± 6.4	BDL
L. antibioticus	L32	0.3	± 0.5	BDL
L. antibioticus	173	33.0	± 2.3	BDL
L. antibioticus	174	1.5	± 0.2	BDL
L. capsici	L12	7.1	± 0.5	BDL
L. capsici	L13	35.0	± 7.1	BDL
L. capsici	L14	9.2	± 1.5	BDL
L. capsici	L31	34.0	± 6.7	BDL
L. enzymogenes	L19	20.0	± 1.2	BDL
L. enzymogenes	L28	2.6	± 0.4	BDL
L. enzymogenes	L29	4.0		BDL
L. enzymogenes	L30	1.6	± 0.2	BDL
L. gummosus	L05	20.0	± 5.3	BDL
L. gummosus	L15	29.0	± 9.6	BDL
L. gummosus	L26	5.2	± 1.4	BDL
L. gummosus	L33	1.5	± 0.1	BDL
